# Perception of urinary biomarker tests among patients referred with suspected urological malignancy

**DOI:** 10.1002/bco2.234

**Published:** 2023-04-03

**Authors:** Nicholas Bullock, Mohamed Mubarak, Ceri Morris, Colette Clements, Clare Geere, Sarah Tidball, Elizabeth Bois, Michael Davies, Jonathan Featherstone, Krishna Narahari, Ian Weeks, Howard Kynaston

**Affiliations:** ^1^ Division of Cancer and Genetics Cardiff University School of Medicine Cardiff UK; ^2^ Department of Urology Cardiff and Vale University Health Board Cardiff UK; ^3^ Urology Research Delivery Team Cardiff and Vale University Health Board Cardiff UK; ^4^ Clinical Innovation Cardiff Cardiff University School of Medicine Cardiff UK; ^5^ Life Sciences Hub Wales Cardiff UK; ^6^ College of Biomedical and Life Sciences Cardiff University Cardiff UK

**Keywords:** bladder cancer, cystoscopy, diagnosis, haematuria, patient perception, urinary biomarker

## Abstract

**Objective:**

To determine the acceptability of a non‐invasive urinary biomarker test in place of conventional flexible cystoscopy for the diagnosis of bladder cancer in patients referred to a Rapid Access Haematuria Clinic (RAHC) with suspected urological malignancy.

**Patients and methods:**

Patients attending a RAHC were recruited to a prospective observational study evaluating a novel urinary biomarker (URO17™) for the detection of bladder cancer and invited to complete a two‐part structured questionnaire. Questions related to demographics, attitudes towards conventional cystoscopy and the minimal acceptable sensitivity (MAS) at which a urinary biomarker would be considered an alternative to flexible cystoscopy both before and after undergoing the procedure.

**Results:**

A total of 250 patients completed the survey; the majority of whom were referred with visible haematuria (75.2%). One hundred seventy‐one (68.4%) would be willing to accept a urinary biomarker in place of cystoscopy, with 59 (23.6%) expressing preference for the biomarker with a MAS as low as 85%. Conversely, 74 patients (29.6%) would not be willing to accept a urinary biomarker, regardless of its sensitivity. A significant number of patients reported a change in MAS after undergoing cystoscopy, with 80 (32.0%) and 16 (6.4%) increasing and decreasing the required value respectively (*P* = 0.001). The greatest increase was seen in the proportion of patients unwilling to accept a urinary biomarker regardless of its sensitivity, rising from 29.6% to 38.4%.

**Conclusions:**

Although many patients attending a RAHC would be willing to accept a urinary biomarker test in place of conventional flexible cystoscopy for the detection of bladder cancer, effective patient, public and clinician engagement will be necessary at all stages of implementation if it is to become an established component of the diagnostic pathway.

## 1 INTRODUCTION

Bladder cancer is the 10th most common cancer worldwide, accounting for approximately 573,000 new cases and 213,000 deaths in 2020.^1^ Approximately 75% of patients have non‐muscle invasive bladder cancer (NMIBC) at the time of diagnosis, which is associated with a risk of both recurrence and progression, thereby mandating risk‐adapted approaches to intravesical treatment and ongoing surveillance.^2^, ^3^ Conversely, those patients found to have muscle invasive bladder cancer (MIBC) or metastases at presentation have a much worse prognosis, with median 5‐year survival estimates of 36.3–69.5% and 4.6%, respectively, despite contemporary treatment.^4^ Timely presentation and investigation are therefore imperative to identifying disease at an early stage and hence optimising outcomes, with studies indicating that improved survival is seen in those with a shorter time to diagnosis.^5^, ^6^

As there is no accepted screening test for bladder cancer, the majority of patients in the United Kingdom (UK) initially present to their General Practitioner in primary care, with subsequent referral to their local urology department for triage, appointment booking and clinical assessment. Visible haematuria is the most common presenting complaint, with a recent large UK multicentre study identifying the prevalence of bladder cancer to be 22.4% in this cohort.^7^ This is acknowledged in the suspected cancer recognition and referral guidelines published by the National Institute for Health and Care Excellence (NICE; NG12), in which patients aged 45 years and over with visible haematuria in the absence of urinary tract infection or aged 60 years and over with non‐visible haematuria and either dysuria or a raised white cell count are recommended to be seen by the receiving urology department within 2 weeks of referral.^8^ Those patients not meeting these criteria are also assessed but within a less prescriptive timeframe in accordance with local urology department protocols.

In most UK institutions patients referred with suspected urological malignancy are assessed in a ‘rapid access’ or ‘one stop’ clinic. Usually, this comprises a clinical assessment together with blood and urine analysis. Upper urinary tract imaging is undertaken with ultrasound and/or computed tomography (CT), and the lower urinary tract is directly visualised using flexible cystoscopy. The personnel responsible varies between centres, but in all cases, the appointment is undertaken by a practitioner (either Urological Surgeon or Specialist Nurse) with sufficient training and expertise in each component so that the patient can be counselled accordingly and either discharged or referred for ongoing investigations and/or treatment as required.

Although flexible cystoscopy has a sensitivity of 98% for the diagnosis of bladder cancer and is the current gold standard test, it is invasive and associated with a reported risk of dysuria (50%), bleeding (19%) and infection (5.5%).^9^ Furthermore, cystoscopy is performed as a component of Secondary Care assessment for which there may be delays in referral or appointment allocation or issues with access for those that reside in remote areas or have limitations in mobility.

Urinary biomarkers have been proposed as a non‐invasive and cost‐effective alternative to cystoscopy for use in both the initial diagnosis and ongoing post‐treatment follow‐up of patients with bladder cancer. Although urinary cytology has been shown to have a high specificity in the diagnosis of high‐grade tumours, it has an overall sensitivity of less than 40%, rendering it unsuitable as a diagnostic tool in isolation.^10^ Several novel urinary biomarkers have been described in the literature and are in varying stages of development, with mechanisms of detection based upon alterations in genomic, transcriptomic, epigenetic or proteomic signatures within samples.^11^ However, whilst such tests are being developed on the premise that they may present a means of avoiding flexible cystoscopy and its associated risks in those with suspected bladder cancer, it is also paramount to establish the attitudes of patients to the use of such a test in their diagnostic pathway. Until now this has not yet been characterised. This prospective questionnaire‐based study therefore sought to establish the attitudes of patients referred to a Rapid Access Haematuria Clinic with suspected urological malignancy to a novel urinary biomarker for the detection of bladder cancer, particularly with respect to the sensitivity required for such a test to become an acceptable alternative to conventional flexible cystoscopy.

## 2 PATIENTS AND METHODS

### 2.1 Patient cohort

Patients attending a RAHC in a single large tertiary referral centre between 28 September 2021 and 8 August 2022 were invited to participate in a prospective observational study exploring the sensitivity and specificity of a urinary biomarker test based on the positivity of cells for keratin 17 when stained with immunohistochemistry, URO17™, as described previously.^12^, ^13^ Those patients who provided informed consent to participate were also invited to complete a structured questionnaire to establish their attitudes towards conventional cystoscopy and a novel urinary biomarker for the detection of bladder cancer, as outlined below.

### 2.2 Questionnaire

A two‐part structured questionnaire was based on a previously published tool used to determine attitudes towards a urinary biomarker for the follow‐up of patients with previously confirmed NMIBC.^14^ Public and patient involvement formed an integral part of the development of patient information and question design to ensure all were appropriately worded, understandable and relevant. The first part was completed by respondents prior to undergoing flexible cystoscopy and comprised questions relating to demographics, attitudes towards cystoscopy and the minimum sensitivity at which a urinary biomarker would be considered an acceptable alternative to flexible cystoscopy [termed minimal acceptable sensitivity (MAS)], as determined using the standard gamble method.^15^ Cystoscopy was defined as having a sensitivity of 98% for the detection of bladder cancer, as previously reported, and MAS was defined as the sensitivity at which patients either expressed a preference for the urinary biomarker or were neutral about accepting either the biomarker or conventional cystoscopy.^9^, ^14^ The second part was completed following flexible cystoscopy and comprised questions relating to the experience of the procedure, as well as whether this resulted in a change in MAS of a urinary biomarker test. Questions relating to patient experience, such as those pertaining to levels of anxiety, discomfort and embarrassment were evaluated using the established method of a five‐point Likert scale.^16^ A full version of the questionnaire is provided in Appendix ^1^.

### 2.3 Statistical analysis

All data analysis was performed using SPSS statistical software version 27 (IBM Corporation, Armonk, New York, USA). Continuous age data were confirmed to be normally distributed and represented as mean [standard deviation (SD)], with statistical comparisons between groups conducted using the one‐way analysis of variance (ANOVA). Categorical data were represented as values and/or percentages, and differences between groups were assessed using the chi‐squared test. The McNemar–Bowker test was used to assess for a significant change in pre‐ and post‐cystoscopy MAS between individual respondents. A value of *p* ≤ 0.05 was used to determine statistical significance.

### 2.4 Ethics and informed consent

This questionnaire‐based research was granted ethical approval as a sub‐component of a larger prospective study exploring the sensitivity and specificity of the URO17™ urinary biomarker test for the diagnosis of bladder cancer (IRAS project ID: SPON 1846‐21, REC reference: 21/PR/0745, granted 10 June 2021). All patients provided written informed consent to participate, and anonymised data were handled in accordance with institutional governance requirements.

## 3 RESULTS

### 3.1 Patient demographics

A total of 250 patients completed the survey. The demographics of the cohort are outlined in Table 1. The mean age of respondents was 65.8 years (SD 11.5 years), with 155 (62.0%) being male and 95 (38.0%) being female. The majority attended the RAHC after being referred with visible haematuria (188; 75.2%), followed by symptomatic non‐visible haematuria (60; 24.0%) and unexplained recurrent urinary tract infection (2; 0.8%). Although most patients had not previously undergone flexible cystoscopy, 62 (24.8%) reported having undergone the procedure at some point previously.

**TABLE 1:** Patient demographics.

Age in years, mean (SD)	65.8 (11.5)
Sex, *n* (%)	
Male	155 (62.0)
Female	95 (38.0)
Highest level of education, *n* (%)	
High school	51 (20.4)
GCSE/O level	56 (22.4)
A level	34 (13.6)
University degree	68 (27.2)
Higher degree	28 (11.2)
Prefer not to say	11 (4.4)
Missing	2 (0.8)
Occupational status, *n* (%)	
Employed—full time	78 (31.2)
Employed—less than full time	15 (6.0)
Retired	143 (57.2)
Unemployed	14 (5.6)
Indication for cystoscopy, *n* (%)	
Visible haematuria	188 (75.2)
Symptomatic non‐visible haematuria	60 (24.0)
Recurrent UTI	2 (0.8)
Previous cystoscopy, *n* (%)	
Yes	62 (24.8)
No	185 (74.0)
Missing	3 (1.2)

Abbreviations: SD, standard deviation; UTI, urinary tract infection.

### 3.2 Pre‐procedural perceptions and experience of flexible cystoscopy

**FIGURE 1:**
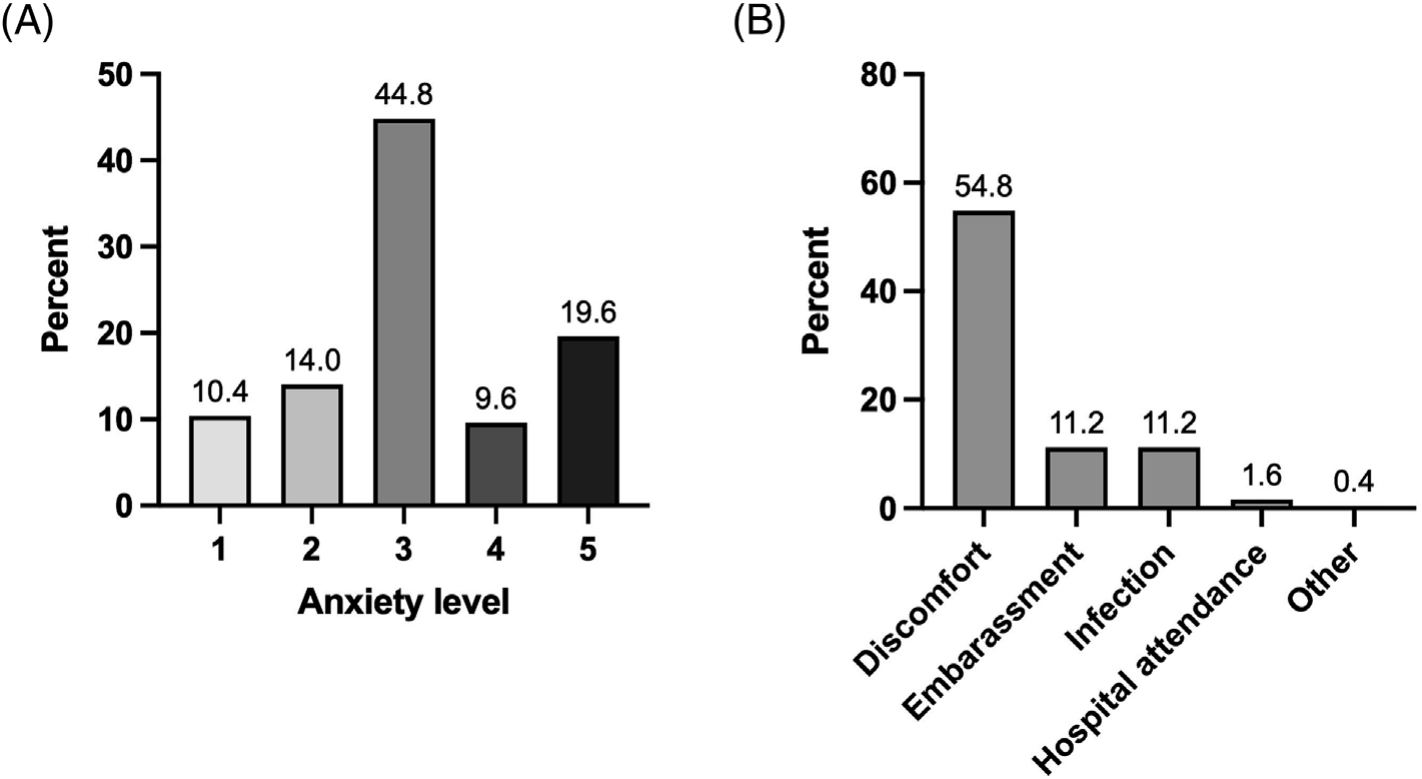
Patient perceptions of conventional diagnostic flexible cystoscopy prior to undergoing the procedure. (A) Self‐reported anxiety level surrounding cystoscopy. 1—not anxious at all to 5—very anxious. (B) The single most significant risk/downside associated with conventional flexible cystoscopy.

Most respondents reported some level of anxiety regarding flexible cystoscopy prior to undergoing the procedure, with 74.0% being somewhat anxious to very anxious, as shown in Figure 1. The greatest perceived downside to flexible cystoscopy was reported to be anticipated discomfort (54.8% of respondents), followed by anticipated embarrassment and risk of infection (11.2% of respondents each).

**FIGURE 2:**
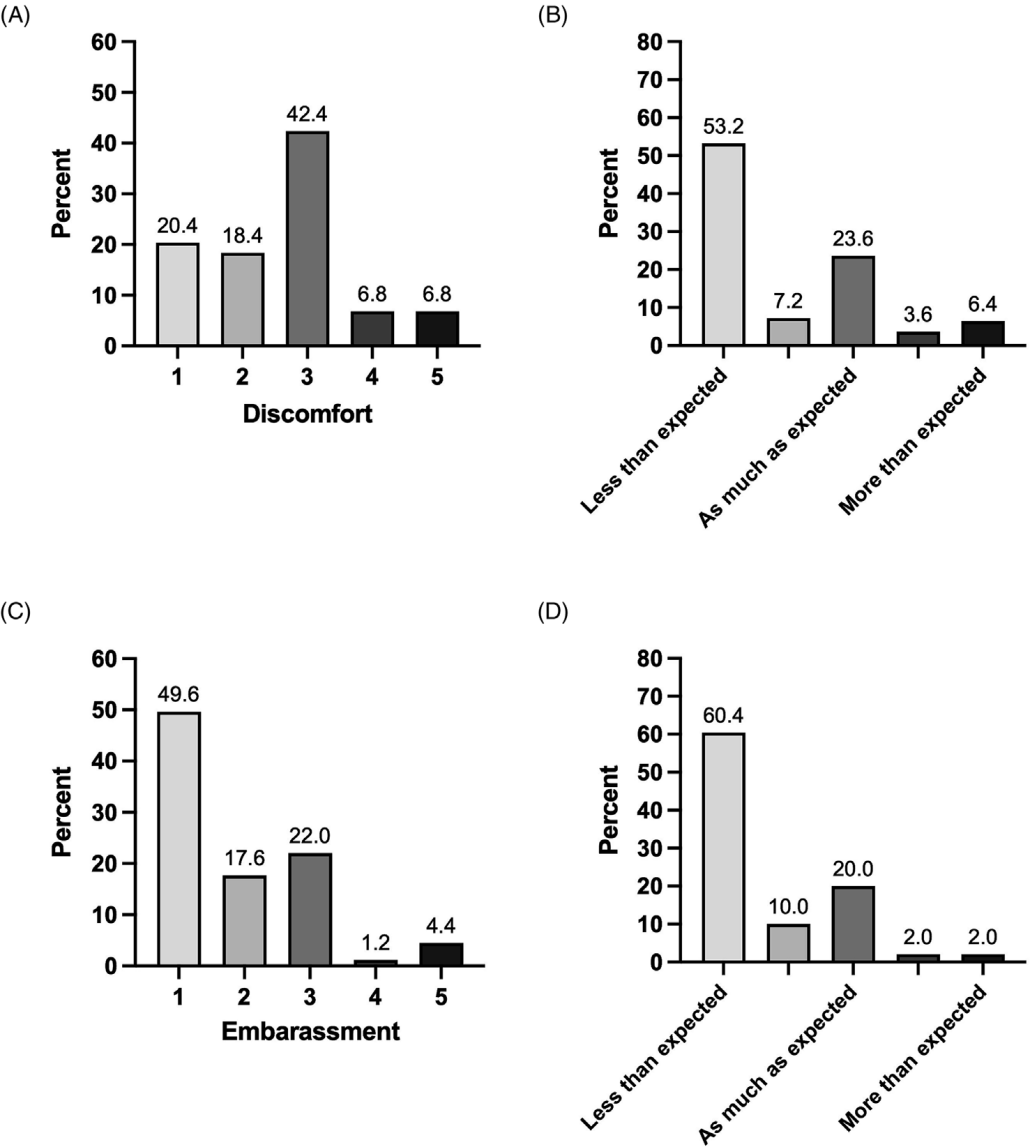
Patient perceptions of conventional diagnostic cystoscopy after undergoing the procedure. (A) Self‐reported discomfort associated with cystoscopy, 1—no discomfort to 5—significant discomfort. (B) Discomfort level compared with expectations held prior to undergoing cystoscopy. (C) Self‐reported embarrassment associated with cystoscopy, 1—not embarrassing at all to 5—significant embarrassment. (D) Embarrassment compared with expectations held prior to undergoing cystoscopy.

Figure 2 demonstrates patient experience of flexible cystoscopy as reported shortly after undergoing the procedure. Although 56.0% of respondents reported some or more discomfort, 20.4% did not experience any discomfort at all, with 53.2% stating that the procedure was less uncomfortable than expected. Similarly, 49.6% of respondents did not report any embarrassment associated with procedure, with 60.4% expressing that it was less embarrassing than expected.

### 3.3 Minimum Acceptable Sensitivity before and after cystoscopy

**FIGURE 3:**
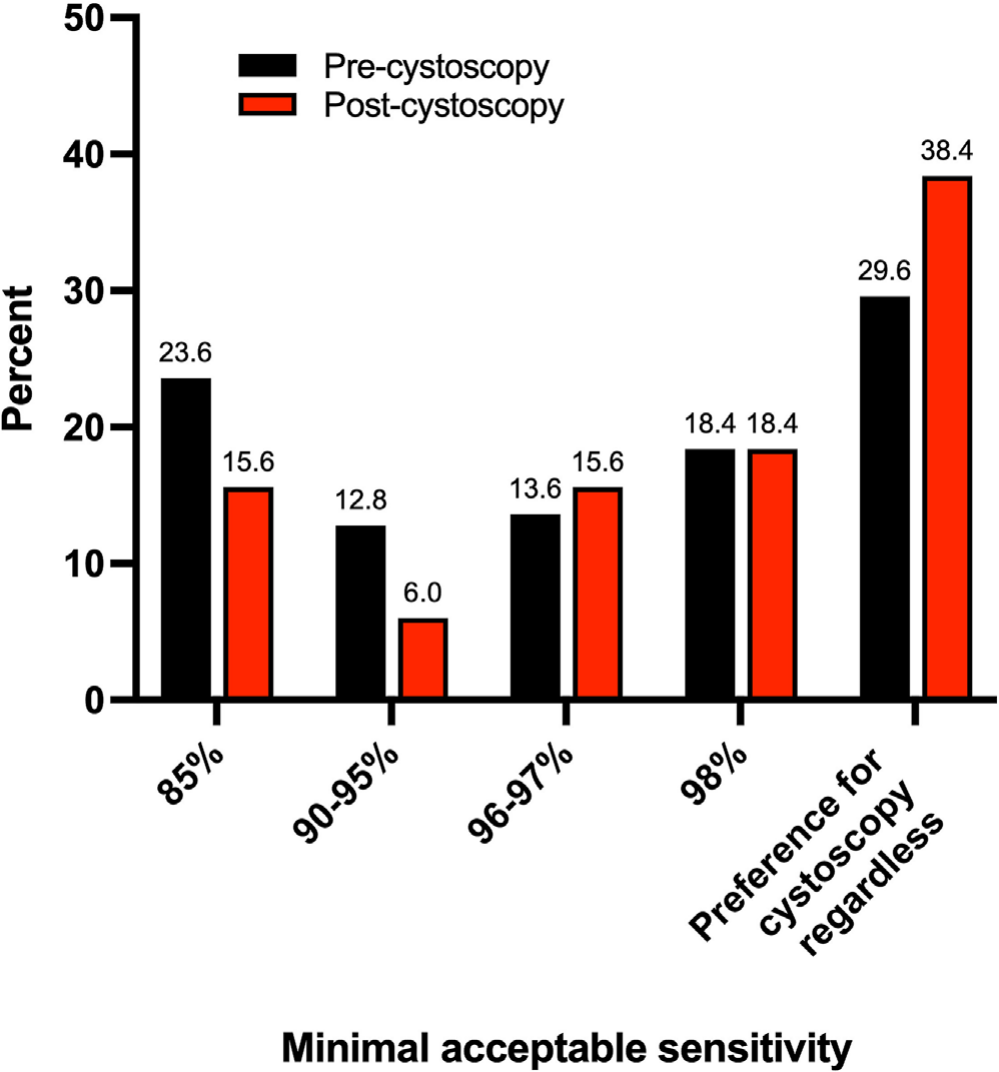
Distribution of minimal acceptable sensitivity values for a urinary biomarker to replace conventional flexible cystoscopy for the diagnosis of bladder cancer, as reported by patients prior to and following flexible cystoscopy.

Figure 3 illustrates the MAS for a urinary biomarker to replace conventional flexible cystoscopy for the diagnosis of bladder cancer as reported before and after undergoing the procedure. Prior to cystoscopy 171 patients (68.4%) were willing to accept a urinary biomarker in place of cystoscopy, with 59 (23.6%) expressing preference for the biomarker with a sensitivity as low as 85% and 46 (18.4%) only willing to accept a biomarker if it had an equivalent sensitivity to cystoscopy. Conversely, 74 patients (29.6%) were not willing to accept a urinary biomarker over conventional cystoscopy, regardless of its sensitivity. Table 2 outlines each demographic variable as stratified according to pre‐procedural MAS. The only variable that was associated with a significant difference in pre‐cystoscopy MAS was the highest reported level of education, with a greater proportion of those patients with a University Degree or higher willing to accept a urinary biomarker test compared with those reporting High School or A Level qualifications (*P* = 0.003). Although a greater proportion of patients who had previously undergone flexible cystoscopy were willing to accept a urinary biomarker test compared with those that had not, differences failed to reach statistical significance (80.3% vs. 66.3%, *P* = 0.057). The reasons for those patients unwilling to accept a urinary biomarker and favouring conventional cystoscopy are given in Table 3. The most commonly expressed reason was a desire to get results for diagnostic tests immediately (79.1%), followed by the reassurance gained through direct contact with the clinical team (73.1%) and the fact that cystoscopy is the current standard diagnostic technique (71.6%).

**TABLE 2:** Differences in minimal acceptable sensitivity for a urinary biomarker to replace conventional flexible cystoscopy for the diagnosis of bladder cancer as reported prior to undergoing the procedure, stratified by patient demographics.

	Minimal acceptable sensitivity					
Variable	85%	90–95%	96–97%	98%	Preference for cystoscopy regardless of sensitivity	*P*
Age in years, mean (SD)	67.8 (12.0)	64.5 (13.0)	64.3 (11.5)	63.9 (11.3)	66.1 (10.7)	0.430
Sex, *n* (%)						
Male	42 (27.6)	19 (12.5)	22 (14.5)	29 (19.2)	40 (26.3)	0.365
Female	17 (18.3)	13 (14.0)	12 (12.9)	17 (18.3)	34 (36.6)	
Highest level of education, *n* (%)						
High school	19 (38.0)	8 (16.0)	4 (8.0)	7 (14.0)	12 (24.0)	0.003
GCSE/O level	15 (27.3)	0 (0.0)	7 (12.7)	8 (14.5)	25 (45.5)	
A level	4 (11.8)	5 (14.7)	7 (20.6)	6 (17.6)	12 (35.3)	
University degree	15 (22.7)	10 (15.2)	8 (12.1)	18 (27.3)	15 (22.7)	
Higher degree	2 (7.1)	9 (32.1)	6 (21.4)	5 (17.9)	6 (21.4)	
Prefer not to say	3 (27.3)	0 (0.0)	2 (18.2)	2 (18.2)	4 (36.6)	
Occupational status, *n* (%)						
Employed—full time	16 (20.5)	10 (12.8)	16 (20.5)	16 (20.5)	20 (25.6)	0.415
Employed—less than full time	2 (14.3)	2 (14.3)	4 (28.6)	1 (7.1)	5 (35.7)	
Retired	37 (26.6)	18 (12.9)	13 (9.4)	28 (20.1)	43 (30.9)	
Unemployed	4 (28.6)	2 (14.3)	1 (7.1)	1 (7.1)	6 (42.9)	
Indication for cystoscopy, *n* (%)						
Visible haematuria	46 (24.9)	22 (11.9)	24 (13.0)	33 (17.8)	60 (32.4)	0.460
Symptomatic non‐visible haematuria	13 (22.4)	9 (15.5)	9 (15.5)	13 (22.4)	14 (24.1)	
Recurrent UTI	0 (0.0)	1 (50.0)	1 (50.0)	0 (0.0)	0 (0.0)	
Previous cystoscopy, *n* (%)						
Yes	21 (34.4)	10 (16.4)	5 (8.2)	13 (21.3)	12 (19.7)	0.057
No	38 (21.0)	21 (11.6)	28 (15.5)	33 (18.2)	61 (33.7)	

Abbreviation: UTI, urinary tract infection.

**TABLE 3:** Reasons for favouring conventional cystoscopy those patients who expressed preference for this diagnostic technique regardless of the sensitivity of a urinary biomarker test, as reported prior to undergoing cystoscopy (*n* = 74). Patients could express more than one option.

Reason	*n* (%)
Established technique and current standard practice	48 (71.6)
Prefer attending hospital	39 (58.2)
Prefer to get results immediately	53 (79.1)
Reassured by contact with the clinical team	49 (73.1)

A significant number of patients reported a change in MAS having undergone cystoscopy, with 80 (32.0%) and 16 (6.4%) increasing and decreasing the sensitivity value required for a urinary biomarker to be sufficient to replace cystoscopy respectively (*P* = 0.001). As shown in Figure 3, the greatest increase was seen in the proportion of patients who would be unwilling to accept a urinary biomarker over conventional cystoscopy regardless of its sensitivity, which rose from 29.6% to 38.4% of the overall cohort.

## 4 DISCUSSION

This is the first study to explore the perceptions of a non‐invasive urinary biomarker as a potential replacement for flexible cystoscopy in the detection of bladder cancer among patients presenting to a rapid access haematuria clinic with suspected urological malignancy. Although undertaken as a component of a broader prospective study of the performance of one particular biomarker, questions did not relate to this specifically and hence may be taken to represent perceptions of the concept as a whole. Over two‐thirds of patients would be willing to accept a biomarker test (68.4%), with the percentage increasing with an increasing level of sensitivity, indicating acceptance to be linked to the diagnostic performance of the test.

However, 29.6% of patients would not be willing to accept a urinary biomarker in place of cystoscopy, regardless of its performance. This proportion increased to 38.4% when patients were asked again having undergone flexible cystoscopy, with a total of 32.0% reporting an increase in MAS, suggesting that their experience of the procedure itself influences their perception of the use of a urinary biomarker test. Indeed, although 44.8% of patients reported some anxiety prior to the procedure, over half felt that cystoscopy was less uncomfortable and embarrassing than they had expected, perhaps influencing the threshold for which the perceived benefits of a urinary biomarker outweigh the risks associated with flexible cystoscopy. This observation indicates that, despite receiving validated information prior to attending their RAHC appointment, many patients' perceptions of cystoscopy did not align with their actual experience, demonstrating a potential need for improved pre‐procedural counselling. As such, optimisation of pre‐procedural patient education regarding both flexible cystoscopy and any alternative diagnostic test would be necessary in order to minimise misunderstanding and better inform decision‐making between options in those being investigated for suspected urological malignancy.

Although there has been extensive research into the development and validation of urinary biomarkers for the detection and follow‐up of bladder cancer, there have been very few studies exploring the perceptions of such tests among those patients for whom they are being developed. The most comparable study is that reported by Tan et al.^14^ Patients with new or recurrent bladder cancer that entered into a multicentre observational study exploring the sensitivity of another biomarker (UroMark; DETECT II, Clinical Trials Number NCT02781428) were invited to complete a structured questionnaire and participate in semi‐structured interviews relating to experience of cystoscopy and perception of a urinary biomarker test. Of the 213 patients who completed the questionnaire component, 163 (76.6%) reported that they would be willing to accept a urinary biomarker test in place of cystoscopy, with the proportion increasing as the sensitivity of the test increased, again indicating a link with performance. Similar to the findings reported here, several patients indicated that they would be unwilling to accept a urinary biomarker in place of cystoscopy, regardless of its sensitivity (21.3%).

Although it is possible to draw some comparisons, there were some notable differences in the patient group that may have resulted in the higher acceptance of a biomarker test than reported here. Firstly, those patients surveyed had an established diagnosis of NMIBC, and as such, the demographic of the cohort differed from that included in the current study, with a greater proportion of males and a higher average age. Perhaps more importantly, the survey sought to evaluate the acceptability of a biomarker to detect disease recurrence in the follow‐up setting, rather than in those first presenting with suspected urological malignancy. It is therefore probable that responses would have differed as a consequence of previous experiences in the diagnostic pathway, including previous exposure to flexible cystoscopy, together with differences in the ideas, concerns and expectations of patients in each group. For example, those undergoing surveillance after an established diagnosis of NMIBC would most likely be concerned about either recurrence or progression of bladder cancer specifically, whereas those referred for initial assessment via the RAHC are likely to be concerned about the full spectrum of urological cancers.

Two other studies have also explored the acceptability of a urinary biomarker as a replacement for cystoscopy in the surveillance setting following the initial treatment of NMIBC. Yossepowitch et al. utilised the standard gamble method to assess the MAS of a urinary biomarker to replace cystoscopy in 200 patients, reporting that 21% would be willing to accept a biomarker for detection of recurrent bladder tumours if it possessed an accuracy of 90–95%, and an additional 75% if it were capable of detecting more than 95%.^17^ Vriesema et al. utilised a similar approach to determine the MAS of a urinary biomarker to replace cystoscopy for surveillance in 102 patients with NMIBC that had undergone at least 1 year of follow‐up.^18^ Although only 11% reported a MAS of 85% or less, 68% reported a MAS of 99%–100%.^18^ In both studies, male sex was associated with a lower MAS.^17^, ^18^ Despite methodological differences again limiting direct comparison with the current study, these findings corroborate the finding that the acceptability of a urinary biomarker is linked to its overall diagnostic performance, regardless of whether this is for use in initial diagnosis or follow up after treatment.

Urinary biomarker tests have several potential advantages when compared with the current RAHC approach for the detection of new bladder cancers. These include minimising the need for invasive procedures and their associated risks, as well as potentially reducing diagnostic delay and streamlining the referral process through their utilisation at an earlier point in the diagnostic pathway, such as at the point of presentation to primary care. Furthermore, the ideal urinary biomarker may also be used to assess disease recurrence after an established diagnosis, detect progression to more invasive disease and potentially predict treatment response.^19^ However, despite these advantages there are a number of well‐established barriers to the implementation of novel diagnostic tests, including the burden of proof in establishing the usefulness of the test (i.e. its analytic and clinical validity), regulatory hurdles, operational viability in terms of the resources required for implementation at scale and proof of cost‐effectiveness within the healthcare setting in which it is to be applied.^20^, ^21^ There may also be resistance among clinicians on account of preference for traditional, gold standard techniques that require professional expertise for analysing and interpreting results.^20^

This study introduces an additional barrier to implementation in the form of patient preference for traditional, gold standard techniques, with 29.6% of respondents expressing that they would not be willing to accept a urinary biomarker in place of conventional diagnostic cystoscopy regardless of its sensitivity, which rose further to 38.4% following the procedure. Therefore, even if large‐scale prospective studies demonstrate a urinary biomarker to possess sufficient performance and cost‐effectiveness to support widespread implementation, its acceptance among clinicians and patients is not guaranteed. Furthermore, among the patient group evaluated in this study, the most widely expressed reason for favouring cystoscopy was the immediacy of results (79.1%), followed closely by reassurance from direct contact with the clinical team (73.1%) and cystoscopy being the current established diagnostic technique (71.6%). As such, these factors must be taken into consideration when designing diagnostic pathways in which biomarkers may play an important part, perhaps through focussing on the development of ‘point‐of‐care’ tests that could be integrated into existing pathways in which patients have some form of contact with healthcare providers that possess relevant expertise.

Alongside high‐quality data on performance and cost‐effectiveness, dissemination of high‐quality education materials for clinicians and patients alike will be key in building confidence in any novel diagnostic technology if it is to become widely utilised. History has taught us that the introduction of techniques and technologies that result in a significant paradigm shift in practice, such as the implementation of laparoscopic techniques for intraabdominal surgery can be slow at first, but once the evidence base and hence clinician and patient confidence builds, they can rapidly become established in routine practice.^22^ Effective patient and public involvement in implementation strategies for a urinary biomarker are therefore paramount if it is to become the standard of care for those referred with suspected bladder cancer.

Despite its prospective design, this study was subject to some limitations. Firstly, a proportion of patients had previously undergone a flexible cystoscopy at some point prior to participation. This may have influenced responses to certain questions, especially those relating to anxiety and the pre‐procedural MAS of the urinary biomarker, as illustrated by the lower proportion of patients expressing initial preference for cystoscopy regardless of biomarker sensitivity than in the overall study population. The exact reason for this is unclear but it is possible that their previous cystoscopy, including any discomfort or complications experienced at the time, may have rendered them more willing to accept a urinary biomarker to avoid going through such an invasive procedure again. Nevertheless, when the proportion of patients having previously undergone cystoscopy was evaluated according to MAS group, the differences did not reach the significance threshold. Secondly, patient and public involvement was utilised in the design of the patient information sheet and questionnaire so as to ensure that all patients were able to comprehend the individual questions asked, regardless of preceding knowledge of the subject area. Furthermore, patients were provided with written information on both flexible cystoscopy and the URO17™ study ahead of their RAHC appointment. However, despite this, it is possible that some questions or concepts may have been misinterpreted, as exemplified by the fact that 52 (20.8%) respondents incorrectly completed the question relating to the most significant downside of flexible cystoscopy. Finally, although data were collected prospectively at the time of attending the RAHC, any associated anxiety surrounding the presence of an underlying malignancy or the flexible cystoscopy procedure itself may have resulted in reporting bias favouring the procedure they were about to receive.

In conclusion, this study demonstrates that over two‐thirds of patients attending a Rapid Access Haematuria Clinic for investigation of suspected urological malignancy would be willing to accept a urinary biomarker test in place of conventional flexible cystoscopy for the diagnosis of bladder cancer, with the percentage increasing with increasing levels of sensitivity of the test. However, a relatively high number would not be willing to accept such a test regardless of its sensitivity, favouring the immediacy of results and reassurance from direct contact with the clinical team associated with conventional cystoscopy. Effective patient, public and clinician education and engagement at all stages of implementation will therefore be a necessary accompaniment to high‐quality data on the performance and cost‐effectiveness of any urinary biomarker test if it is to become an established component of the diagnostic pathway for those referred with suspected urological cancer.

## AUTHOR CONTRIBUTIONS

Nicholas Bullock, Ceri Morris, Jonathan Featherstone, Krishna Narahari and Howard Kynaston were involved in study initiation and questionnaire design. Nicholas Bullock, Ceri Morris, Colette Clements, Clare Geere, Sarah Tidball, Elizabeth Bois, Jonathan Featherstone, Krishna Narahari and Howard Kynaston were responsible for patient recruitment and data collection. Nicholas Bullock, Mohamed Mubarak, Michael Davies, Jonathan Featherstone, Krishna Narahari, Ian Weeks and Howard Kynaston were involved in data analysis, interpretation and manuscript preparation. All authors participated in manuscript review and revision prior to submission.

## CONFLICT OF INTEREST STATEMENT

All authors are involved in an ongoing prospective study exploring the sensitivity of the URO17™ urinary biomarker test for the diagnosis of bladder cancer in patients referred to a RAHC with suspected urological malignancy (IRAS project ID: SPON1846‐21, REC reference: 21/PR/0745, granted 10 June 2021).

## Supporting information




**Data S1.** Supporting Information
