# Supplementary material for: Perception of urinary biomarker tests among patients referred with suspected urological malignancy
Source: BJUI Compass. 2023 Apr 3;4(4):446–54. doi: 10.1002/bco2.234 (PMC10268572; doi:10.1002/bco2.234)
Supplement: Supplementary file 1 — Data S1. Supporting Information [file BCO2-4-446-s001.docx]

Acceptability of Flexible Cystoscopy for the investigation of suspected bladder cancer

**Participant information**

Whilst blood in the urine (known as haematuria) or other urinary symptoms such as burning or stinging when passing water (known as dysuria) are often caused by infection, inflammation or stones, in a small number of patients they may also be caused bladder cancer. It is therefore important that people with these symptoms are investigated promptly using tests that are effective and pose minimal risk of complications so as to enable treatment to be commenced. Currently the most effective diagnostic test for bladder cancer is to directly look inside the bladder using a small flexible telescope under local anaesthetic, known as flexible cystoscopy. This gives the person carrying out the examination an immediate result and enables rapid progression to further investigations and treatment if required. However, flexible cystoscopy is also an invasive test that is associated with a risk of discomfort, bleeding and infection.

Research has shown that flexible cystoscopy has a sensitivity of 98%, which means that it is able to detect bladder cancer in 98 out of 100 patients that have the disease [1]. In recent years scientists have developed a number of different tests that aim to diagnose bladder cancer from blood or urine samples without the need for flexible cystoscopy. It has been proposed that if effective, they may present an alternative to flexible cystoscopy. One of these tests, known as Uro17, is currently being trialled at the University Hospital of Wales.

As someone who is attending the Department of Urology for a flexible cystoscopy, we are interested to hear your views on the procedure itself, and any alternative non-invasive tests that may become available in the future. If you are willing to participate, please sign the consent form and complete the first part of the questionnaire PRIOR to the procedure. Please then complete the second part of the questionnaire AFTER you have had the procedure.

Thank you for participating in this research. All of your responses will be anonymised and will have no bearing on the treatment you receive. You are free to withdraw your consent at any time.

**References:**

1. Blick, C.G., et al., *Evaluation of diagnostic strategies for bladder cancer using computed tomography (CT) urography, flexible cystoscopy and voided urine cytology: results for 778 patients from a hospital haematuria clinic.* BJU international, 2012. **110**(1): p. 84-94.

**Part 1**

To be completed prior to undergoing flexible cystoscopy

**1.1 Information about you**

1. ID number (to be entered by recruiting personnel):

­­­­­­

1. What is your sex (please tick)

Male

Female

Prefer not to say

1. What is your highest level of education (please tick the most appropriate response)?

**OPTIONAL QUESTION**

High school

GCSE / O Level

A Level

University degree

Higher degree (PhD etc)

Prefer not to say

1. What best describes your current occupational status (please tick the most appropriate response)?

Retired

Currently unemployed

Employed – full time

Employed – part time

**1.2 Clinical information**

1. Have you ever had a flexible cystoscopy before?

Yes

No

1. What is the reason for attending for a flexible cystoscopy today (please tick the single most appropriate response)?

Blood in urine that you or someone else has seen with the naked eye (visible haematuria)

Blood in urine that has been detected on a urine sample but cannot be seen with the naked eye, WITHOUT any other symptoms (known as asymptomatic non-visible haematuria)

Blood in urine that has been detected on a urine sample but cannot be seen with the naked eye, WITH other lower urinary tract symptoms such as burning/stinging when passing water (known as symptomatic non-visible haematuria)

Recurrent urinary tract infections

An abnormality in your bladder that has been seen on a previous scan of your abdomen (for example on an ultrasound or CT scan)

Other (please state):

**1.3 Perspective on cystoscopy versus a non-invasive urine test for the diagnosis of bladder cancer**

1. Please indicate your anxiety about undergoing the procedure today on the scale below

| Not anxious at all |  | Somewhat anxious |  | Very anxious |
| --- | --- | --- | --- | --- |
| 1 | 2 | 3 | 4 | 5 |

1. What do you see as the most significant downside to flexible cystoscopy (please tick the most appropriate response)?

Anticipated discomfort

Anticipated embarrassment / lack of dignity

Risk of infection

Requirement to specifically attend hospital and have time away from work/other activities

Other (please state):

1. Neither flexible cystoscopy nor a non-invasive urinary test will be able to diagnose all bladder cancers. Whilst it is known that cystoscopy is able to detect bladder cancer in 98 out of 100 cases, 2 out of 100 will therefore be missed. The following questions relate to your preference of a urine test over flexible cystoscopy based on its proven ability to detect a certain percentage of cancers.
2. If the urine test will miss 15 out of 100 bladder cancers (versus 2 out of 100 for cystoscopy), would you prefer the urinary test or cystoscopy?

| Prefer urinary test | Neutral | Prefer cystoscopy |
| --- | --- | --- |
| 1 | 2 | 3 |

1. If the urine test will miss 10 out of 100 bladder cancers (versus 2 out of 100 for cystoscopy), would you prefer the urinary test or cystoscopy?

| Prefer urinary test | Neutral | Prefer cystoscopy |
| --- | --- | --- |
| 1 | 2 | 3 |

1. If the urine test will miss 8 out of 100 bladder cancers (versus 2 out of 100 for cystoscopy), would you prefer the urinary test or cystoscopy?

| Prefer urinary test | Neutral | Prefer cystoscopy |
| --- | --- | --- |
| 1 | 2 | 3 |

1. If the urine test will miss 6 out of 100 bladder cancers (versus 2 out of 100 for cystoscopy), would you prefer the urinary test or cystoscopy?

| Prefer urinary test | Neutral | Prefer cystoscopy |
| --- | --- | --- |
| 1 | 2 | 3 |

1. If the urine test will miss 5 out of 100 bladder cancers (versus 2 out of 100 for cystoscopy), would you prefer the urinary test or cystoscopy?

| Prefer urinary test | Neutral | Prefer cystoscopy |
| --- | --- | --- |
| 1 | 2 | 3 |

1. If the urine test will miss 4 out of 100 bladder cancers (versus 2 out of 100 for cystoscopy), would you prefer the urinary test or cystoscopy?

| Prefer urinary test | Neutral | Prefer cystoscopy |
| --- | --- | --- |
| 1 | 2 | 3 |

1. If the urine test will miss 3 out of 100 bladder cancers (versus 2 out of 100 for cystoscopy), would you prefer the urinary test or cystoscopy?

| Prefer urinary test | Neutral | Prefer cystoscopy |
| --- | --- | --- |
| 1 | 2 | 3 |

1. If the urine test will miss 2 out of 100 bladder cancers (the same as for cystoscopy), would you prefer the urinary test or cystoscopy?

| Prefer urinary test | Neutral | Prefer cystoscopy |
| --- | --- | --- |
| 1 | 2 | 3 |

1. What other factors would influence your decision to opt for either flexible cystoscopy or a non-invasive urine test in preference of the other (please select all that apply)?

| Prefer flexible cystoscopy | | Prefer non-invasive urine test | |
| --- | --- | --- | --- |
| It is an established technique and currently standard practice |  | It avoids the possible side effects of cystoscopy (discomfort, bleeding, infection) |  |
| I prefer coming to the hospital for diagnostic tests |  | It is less embarrassing than flexible cystoscopy |  |
| I prefer to get the results of the test immediately |  | It does not require a hospital visit and time away from work/other activities |  |
| I am reassured by contact with the clinical team during the procedure |  | The idea of undergoing flexible cystoscopy makes me anxious/worried |  |
| Other (please state) | | Other (please state) | |

1. Where/how would it be most convenient for you to provide the urine sample for testing (please select most appropriate response)?

At the hospital

At my local GP surgery

At home, with the sample then sent via post to the urology department

**Part 2**

To be completed after undergoing flexible cystoscopy

**2.1 Experience during flexible cystoscopy**

1. Having now undergone flexible cystoscopy, please indicate your level of discomfort during the procedure using the scale below:

| No discomfort |  | Some discomfort |  | Significant discomfort |
| --- | --- | --- | --- | --- |
| 1 | 2 | 3 | 4 | 5 |

1. Please indicate to what extent the discomfort you experienced met your preceding expectations:

| Less discomfort than expected |  | As much discomfort as expected |  | More discomfort than expected |
| --- | --- | --- | --- | --- |
| 1 | 2 | 3 | 4 | 5 |

1. Please indicate your level of embarrassment during the procedure using the scale below:

| Not embarrassing at all |  | Some embarrassment |  | Significant embarrassment |
| --- | --- | --- | --- | --- |
| 1 | 2 | 3 | 4 | 5 |

1. Please indicate to what extent the embarrassment you experienced met your preceding expectations:

| Less embarrassment than expected |  | As much embarrassment as expected |  | More embarrassment than expected |
| --- | --- | --- | --- | --- |
| 1 | 2 | 3 | 4 | 5 |

**2.2 Perspective on cystoscopy versus a non-invasive urine test for the diagnosis of bladder cancer**

1. It is possible that having now undergone the procedure, your attitude towards a non-invasive urinary test may have changed. The following questions therefore again relate to your preference of a urine test over flexible cystoscopy based on its proven ability to detect a certain percentage of cancers.
   1. If the urine test will miss 15 out of 100 bladder cancers (versus 2 out of 100 for cystoscopy), would you prefer the urinary test or cystoscopy?

| Prefer urinary test | Neutral | Prefer cystoscopy |
| --- | --- | --- |
| 1 | 2 | 3 |

- 1. If the urine test will miss 10 out of 100 bladder cancers (versus 2 out of 100 for cystoscopy), would you prefer the urinary test or cystoscopy?

| Prefer urinary test | Neutral | Prefer cystoscopy |
| --- | --- | --- |
| 1 | 2 | 3 |

- 1. If the urine test will miss 8 out of 100 bladder cancers (versus 2 out of 100 for cystoscopy), would you prefer the urinary test or cystoscopy?

| Prefer urinary test | Neutral | Prefer cystoscopy |
| --- | --- | --- |
| 1 | 2 | 3 |

- 1. If the urine test will miss 6 out of 100 bladder cancers (versus 2 out of 100 for cystoscopy), would you prefer the urinary test or cystoscopy?

| Prefer urinary test | Neutral | Prefer cystoscopy |
| --- | --- | --- |
| 1 | 2 | 3 |

- 1. If the urine test will miss 5 out of 100 bladder cancers (versus 2 out of 100 for cystoscopy), would you prefer the urinary test or cystoscopy?

| Prefer urinary test | Neutral | Prefer cystoscopy |
| --- | --- | --- |
| 1 | 2 | 3 |

- 1. If the urine test will miss 4 out of 100 bladder cancers (versus 2 out of 100 for cystoscopy), would you prefer the urinary test or cystoscopy?

| Prefer urinary test | Neutral | Prefer cystoscopy |
| --- | --- | --- |
| 1 | 2 | 3 |

- 1. If the urine test will miss 3 out of 100 bladder cancers (versus 2 out of 100 for cystoscopy), would you prefer the urinary test or cystoscopy?

| Prefer urinary test | Neutral | Prefer cystoscopy |
| --- | --- | --- |
| 1 | 2 | 3 |

- 1. If the urine test will miss 2 out of 100 bladder cancers (the same as for cystoscopy), would you prefer the urinary test or cystoscopy?

| Prefer urinary test | Neutral | Prefer cystoscopy |
| --- | --- | --- |
| 1 | 2 | 3 |

Thank you for taking the time to complete this questionnaire.
